# Unraveling the evolutionary origin of the *P5CS* gene: a story of gene fusion and horizontal transfer

**DOI:** 10.3389/fmolb.2024.1341684

**Published:** 2024-04-17

**Authors:** João Pedro Carmo Filgueiras, Marcel Zámocký, Andreia Carina Turchetto-Zolet

**Affiliations:** ^1^ Graduate Program in Genetics and Molecular Biology, Department of Genetics, Institute of Biosciences, Federal University of Rio Grande do Sul (UFRGS), Porto Alegre, Brazil; ^2^ Laboratory of Phylogenomic Ecology, Institute of Molecular Biology, Slovak Academy of Sciences, Bratislava, Slovakia

**Keywords:** gene duplication, gene fusion, proline, stress response, gene family evolution

## Abstract

The accumulation of proline in response to the most diverse types of stress is a widespread defense mechanism. In prokaryotes, fungi, and certain unicellular eukaryotes (green algae), the first two reactions of proline biosynthesis occur through two distinct enzymes, γ-glutamyl kinase (GK E.C. 2.7.2.11) and γ-glutamyl phosphate reductase (GPR E.C. 1.2.1.41), encoded by two different genes, *ProB* and *ProA*, respectively. Plants, animals, and a few unicellular eukaryotes carry out these reactions through a single bifunctional enzyme, the Δ^1^-pyrroline-5-carboxylate synthase (P5CS), which has the GK and GPR domains fused. To better understand the origin and diversification of the *P5CS* gene, we use a robust phylogenetic approach with a broad sampling of the *P5CS*, *ProB* and *ProA* genes, including species from all three domains of life. Our results suggest that the collected *P5CS* genes have arisen from a single fusion event between the *ProA* and *ProB* gene paralogs. A peculiar fusion event occurred in an ancestral eukaryotic lineage and was spread to other lineages through horizontal gene transfer. As for the diversification of this gene family, the phylogeny of the *P5CS* gene in plants shows that there have been multiple independent processes of duplication and loss of this gene, with the duplications being related to old polyploidy events.

## 1 Introduction

The accumulation of proline in response to various stress types is a widespread defense mechanism among bacteria, yeasts, plants, and marine invertebrates (Ahad and Syiem, 2021; Li et al., 2021; Meng et al., 2021; Wang et al., 2021). The proline biosynthetic pathway, using glutamate as substrate, is conserved in all living organisms. This pathway occurs through three irreversible enzymatic reactions (Fichman et al., 2015). In prokaryotes, fungi, and some unicellular eukaryotes (e.g., green algae), the γ-glutamyl kinase (GK E.C. 2.7.2.11 — encoded by *ProB* gene) and γ-glutamyl phosphate reductase (GPR E.C. 1.2.1.41 — encoded by *ProA* gene), catalyze the first two reactions of proline biosynthesis, converting glutamate in glutamate-5-semialdehyde (GSA) (Baich, 1969). In plants, animals and other unicellular eukaryotes (e.g., oomycete *Phytophthora sojae* and photosynthetic diatom *Phaeodactylum tricornutum*), a bifunctional enzyme, Δ^1^-pyrroline-5-carboxylate synthase (P5CS), with the domains GK and GPR, catalyzes these first two reactions (Smith et al., 1980; Hu et al., 1992; Fichman et al., 2015). An alternative substrate that can be used for proline synthesis is ornithine. The enzyme ornithine aminotransferase (OAT) catalyzes a reversible transfer of δ-amino group of ornithine to α-ketoglutarate, producing GSA and glutamate, respectively (Delauney and Verma, 1993). Because it is a reversible reaction, the GSA produced by the enzymes GK and GPR can also be used to synthesize ornithine (Ginguay et al., 2017). However, this enzyme seems to be mainly involved in the catabolism of arginine in bacteria (Fichman et al., 2015) and in plants (Funck et al., 2008). Ornithine can also be directly converted into proline via the ornithine cyclodeaminase (OCD) enzyme (Trovato et al., 2001), but this route is found only in some prokaryotic groups (Fichman et al., 2015).

The enzyme γ-GK and its homologous portion of P5CS in plants is one of the feedback points of biosynthesis, in which proline will act as a competitive inhibitor (Pérez-Arellano et al., 2010; Forlani et al., 2024). In humans, the native form of P5CS is insensitive to proline or ornithine. Still, in the gut, *P5CS* undergoes an alternative splicing process, encoding an enzyme shortened by two amino acids, making the P5CS allosterically inhibited by ornithine (Hu et al., 1999). In addition to the GK domain, γ-GK enzymes have another domain called PUA (pseudouridine synthase and archeosine transglycosylase). The PUA domain is usually found associated with enzymes that catalyze post-transcriptional modifications in tRNA and rRNA. But the PUA domain is not present in all γ-GK enzymes, around 20% of bacteria and yeasts do not naturally have it, suggesting that the presence of the PUA domain is not essential for the functioning of the γ-GK enzyme (Pérez-Arellano et al., 2007).

The two domains of the *P5CS* gene have correspondence with the *ProA* and *ProB* genes, which may denote a common origin between them (Supplementary Figure S1). For example, the GK portion of the *Vigna aconitifolia P5CS* gene has a 55.3% similarity with the *E. coli ProB* gene. In comparison, the *P5CS* GPR domain has a 57.9% similarity with the *ProA* gene, slightly more conserved (Turchetto-Zolet et al., 2009). Even *ProA* and *ProB* genes, which have different catalytic activities, appear to have originated from a single ancestral gene due to the high level of similarity (42.4%) in their sequence and their three-dimensional structure (Rai and Penna, 2013). The P5CS in humans is localized in the mitochondrial inner membrane (Hu et al., 2008). In contrast to P5CS in plants, it was believed that it was present in the cytoplasm and, in stress situations, also in the chloroplast (Szèkely et al., 2008). However, a new study showed that P5CS is located only in the cytoplasm (Funck et al., 2020). In the case of yeast, the γ-GK and γ-GPR are present in the cytoplasm (Takagi, 2008).

In angiosperms, it is widespread to find at least two gene paralogs that encode the P5CS enzyme, which possibly arose from multiple independent processes of duplication (Turchetto-Zolet et al., 2009; Ma et al., 2022). Gene duplication is even found in bacteria, with some species having two genes that express the enzyme γ-GK (Brill et al., 2011). In contrast, mammals have a single *P5CS* gene, with two different isoforms generated by the alternative splicing process, as already mentioned (Hu et al., 1999).

A previous study performed an evolutionary analysis of the *P5CS, ProA,* and *ProB* genes and found that bifunctional P5CSs fall into clades distinct from the monofunctional orthologs (Fichman et al., 2015). The authors proposed that the origin of the *P5CS* gene probably occurred from the fusion of the *ProA* and *ProB* genes. However, due to small sampling, it is still unclear whether this was a single event that spread via horizontal gene transfer (HGT) or occurred multiple times independently in the ancestor of the plants, animals, and unicellular eukaryotic lineages that have *P5CS*. The fusion of metabolic enzymes may arise due to the metabolic channeling of substrates (Enright et al., 1999), in addition to ensuring that the domains are co-located and co-expressed (Lees et al., 2016). Horizontal gene transfer (HGT) also known as lateral gene transfer (LGT) is a phenomenon that is regularly observed during routine genomic analyses (Syvanen, 2012; Daubin and Szöllösi, 2016). It was demonstrated that mainly several bacteria can transfer parts of plasmid DNA directly into cells of plants, fungi and mammals via the conjugation mechanism (Mizuta et al., 2012). Further, it was detected that mainly host-parasite interactions promote HGT events based on various transposons (Gilbert et al., 2010). There were particular examples of such HGTs where transferred structural genes acquired similar function in recipient organisms (e.g., Zámocký et al., 2012).

With a broad sampling of available DNA sequences and annotated species (526 *P5CS* sequences from 370 species, 736 *ProB* sequences from 648 species, and 641 *ProA* sequences from 621 species), the main goal of this study is to trace the evolutionary history of the *P5CS* gene family and uncover its origin and diversification. Based on these data, we try to answer the following questions: 1) What is the evolutionary relationship between the *P5CS*, *ProA,* and *ProB* genes? 2) Did the *P5CS* gene appear in eukaryotic species after the fusion of *ProA* and *ProB* genes in a single event, or were they independent events throughout the evolution of these organisms? 3) Have some species lost the *P5CS* gene, totally or partially? 4) What evolutionary mechanisms promoted the diversification of the P5CS gene in plants?

## 2 Materials and methods

### 2.1 Data sources

We used BLASTp searches in Ensembl (https://www.ensembl.org/index.html), Phytozome v. 12.1 (https://phytozome.jgi.doe.gov/pz/portal.html) and Metazome v.3.2 (https://metazome.jgi.doe.gov/pz/portal.html) databases to search for *P5CS*, *ProB* and *ProA* coding sequences (CDS) from 1,028 species, of which 693 are eukaryotes and 335 prokaryotes. We used the following queries sequences for the BLAST searches: *P5CS2* gene of *Arabidopsis thaliana* for plants species, the *Homo sapiens P5CS* gene for animals, the *ProB* and *ProA* genes of *E. coli* for bacteria and archaea species, and the *ProB* and *ProA* genes of *Saccharomyces cerevisiae* for fungi species. We considered the default parameter of each database for the e-value threshold. We evaluated the e-value, sequence length and the presence of the domains (GK and/or GPR) to select the sequence for our analyses. For the loci with multiple isoforms predicted, we selected the primary isoform following the information available on the databases used.

We used hmmscan (https://www.ebi.ac.uk/Tools/hmmer/search/hmmscan) to check the integrity and domains in the collected sequences. We retrieved the taxonomic information present in Supplementary Table S1 from the List of Prokaryotic names with Standing in Nomenclature—(LPSN- https://www.bacterio.net/), Catalog of Life (https://www.catalogueoflife.org) and Mycobank databases (https://www.mycobank.org/). We also used TargetP (Armenteros et al., 2019), to search for signal sequences in the *P5CS* gene.

### 2.2 Multiple sequence alignment and phylogenetic analysis

The amino acid sequences were aligned in MAFFT with the L-INS-i algorithm (Katoh et al., 2019). We used MUSCLE (Edgar, 2004), implemented in the MEGA X (Kumar et al., 2018) for alignments of nucleotide sequences (Coding sequence — CDS) from plant species. We removed the extra domains found in some sequences before aligning and kept just the GK, GPR, and PUA domains. The sequences were aligned separately for each gene, thus having an alignment for *ProA*, *ProB* and *P5CS*. The alignments were evaluated and adjusted manually, removing the misaligned portions. For the analysis of the GK and GPR domains, the alignment of the P5CS gene was first divided into two parts, according to its two catalytic domains (Fichman et al., 2015). Then the portion of the *P5CS* gene corresponding to the GK domain was aligned with the *ProB* gene, and the portion of *P5CS* corresponding to the GPR domain was aligned with the *ProA* gene (Supplementary Figure S2).

We performed the phylogenetic analysis for each gene (*ProB*, *ProA* and *P5CS*) separately, including all the sequences collected from each type. For the *ProB* gene, the region of domain PUA has not been considered in the analysis. A phylogeny of the *P5CS* gene only with the species belonging to Viridiplantae was also estimated to better understand gene duplication/loss patterns in this group. And finally, we also resolved the phylogenetic analysis for the GK and GPR domain separately. So, in total, the phylogenetic analysis was performed on six data sets. The details of each analysis are described in Table 1.

**TABLE 1:** Details of the datasets analyzed in the study.

Dataset	Description	N° of sequences	N° of species	Method	UFBoot	Data type	N° of sites	Model
Viridiplantae P5CS	P5CS from plants and algae	183	082	ML	10,000	Nucleotide	2139	GTR + F + R5
All P5CS	All sequences collected from P5CS	533	373	ML	10,000	Aminoacid	652	Q.insect + R9
All ProB	All sequences collected from ProB	732	647	ML	10,000	Aminoacid	231	Q.pfam + I + R10
All ProA	All sequences collected from ProA	641	622	ML	10,000	Aminoacid	383	LG + F + I + R10
GPR domain	All ProA sequences, their homologous domain in P5CS sequences and Putative sequences of P5CS	1193	1008	ML	10,000	Aminoacid	372	LG + F + I + R10
GK domain	All ProB sequences, their homologous domain in P5CS sequences and Putative sequences of P5CS	1278	1012	ML	10,000	Aminoacid	212	Q.pfam + I + R10

We used the Maximum Likelihood (ML) method to estimate the phylogenetic trees in IQTREE v2.2.2 (Minh et al., 2020) with 10,000 ultrafast bootstrap approach (UFBoot) (Minh et al., 2013). The best substitution model for nucleotides was determined by ModelFinder (Kalyaanamoorthy et al., 2017), included in the IQTree. We ran IQtree three times for each alignment and chose the phylogeny with the highest likelihood. All trees were viewed and edited in FigTree.

### 2.3 Synteny analysis

For trypanosomatids and green algae, we performed a synteny analysis around the *P5CS* gene, with the web-software SimpleSynteny (Veltri et al., 2016), looking for possible rearrangements that might have occurred in this region. We used as reference a species that has the complete *P5CS* gene (*Trypanosoma theileri* for trypanosomatids; *Coccomyxa subellipsoidea* C-169 for algae) and we searched for four genes upstream and four genes downstream to *P5CS* and *ProB*. The complete genomes were acquired via NCBI, and for trypanosomatids, we used the following parameters: BLAST E-value Threshold of 0.0001 and Minimum Query Coverage cutoff of 15%. This software did not get a good resolution for algae, so we just used it as a visualization tool. The BLAST search was performed via Ensembl, with the default parameters and choosing the hit with the highest E-value.

## 3 Results

### 3.1 Global identification of *P5CS*, *ProA* and *ProB* genes

We retrieved 526 *P5CS* sequences from 370 species, 736 *ProB* sequences from 648 species, and 641 *ProA* sequences from 621 species. Table 2 shows the distribution of these sequences in the major phyla and the number of species with gene duplications. Of the 183 plant sequences, the presence of a signal peptide was detected in only one sequence (poale_hvu2) with a 0.67 probability of being transported to the chloroplast. Of the 272 animal sequences, 203 have a signal peptide for transport to mitochondria with a mean probability of 0.85, being found even in the two species of choanoflagellates, 64 sequences have no signal peptide and five have a signal peptide, but for a non-specific cellular sublocation (SP) (Supplementary Table S2).

**TABLE 2:** Distribution of sequences collected in the major phyla and the number of species with gene duplications.

Group	P5CS			ProB - GK			ProA - GPR		
	Species	Species/N° of genes	Total seq	Species	Species/N° of genes	Total seq	Species	Species/N° of genes	Total seq
**Algae**	020	15 sp/1 gene	025	035	22 sp/1 gene	056	010	08 sp/1 gene	012
					10 sp/2 genes				
		05 sp/2 genes			01 sp/5 genes			02 sp/2 genes	
					01 sp/3 genes				
					01 sp/6 genes				
**Animals**	232	191 sp/1 gene	275	---	---	---	---	---	---
		39 sp/2 genes							
		02 sp/3 genes							
**Plants**	079	20 sp/1 gene	180	---	---	---	---	---	---
		41 sp/2 genes							
		06 sp/3 genes							
		08 sp/4 genes							
		01 sp/5 genes							
		01 sp/6 genes							
		01 sp/7 genes							
		01 sp/10 genes							
**Protozoa**	027	21 sp/1 gene	34	034	23 sp/1 gene	60	028	26 sp/1 gene	034
					05 sp/2 genes				
		05 sp/2 genes			02 sp/3 genes			01 sp/2 genes	
					02 sp/4 genes				
		01 sp/3 genes			01 sp/5 genes			01 sp/6 genes	
					01 sp/8 genes				
**Eubacteria**	---	---	---	283	274 sp/1 gene	292	280	275 sp/1 gene	285
					09 sp/2 genes			05 sp/2 genes	
**Cyanobacteria**	---	---	---	027	27 sp/1 gene	027	027	27 sp/1 gene	027
**Archaea**	---	---	---	024	24 sp/1 gene	024	024	24 sp/1 gene	024
**Unicellular fungi**	---	---	---	042	35 sp/1 gene	050	042	41 sp/1 gene	043
					06 sp/2 genes			01 sp/2 genes	
					01 sp/3 genes				
**Fungus-like**	012	12 sp/1 gene	012	001	01 sp/1 gen	001	001	01 sp/1 gen	001
**Multicellular fungi**	---	---	---	202	198 sp/1 gene	226	209	203 sp/1 gene	215
					10 sp/2 genes				
					01 sp/3 genes				
					01 sp/4 genes			06 sp/2 genes	
					01 sp/5 genes				
					01 sp/6 genes				
**Total**	370	---	526	648	---	736	620	---	641

For some taxa, the BLAST search had no significant results for any of the three genes (Supplementary Table S1). Given that our BLAST analysis covers multiple species within these taxa, we believe that these groups have lost the genes *ProB*/*ProA* and *P5CS*, rather than it being an assembly error. Among these taxa are the phylum Microsporidia (29 species sampled), the classes Aconoidasida (28 species) and Dictyosteliomycetes (4 species), the order Hymenostomatida (3 species), the family Hexamitidae (3 species), Onchocercidae (3 species) and the genus *Entamoeba* (4 species). Interestingly, these groups mentioned are mandatory or facultative parasites.

Some lineages of single-celled eukaryotes (e.g., Trypanosomatida, Bacillariophyceae, Oomycetes and *Acanthamoeba*) have the bifunctional enzyme P5CS and not the genes *ProB* and *ProA*. And all the Eumycota species sampled here have the *ProB* and *ProA* genes, not the *P5CS*. Therefore, having the P5CS gene is not a characteristic linked to multicellularity. This paper shows five green algae samples with the *ProB* gene and the bifunctional enzyme P5CS. We drew attention to 48 sequences (the most belonging to groups like Chlorophyceae, Oomycetes, and Trypanosomatida) containing only GK or GPR domains. Initially, these single-domain sequences were categorized, like all the others, as being *ProB* or *ProA* genes. However, preliminary analyses showed that these 48 sequences had some divergence from the *ProB*/*ProA* genes (data not shown). For this reason, these sequences were only included in the domain phylogenies. Furthermore, in particular species within these taxa, we found the complete *P5CS* gene (with the GK and GPR domains) in their genome. Based on this, we hypothesized that these 48 sequences with a single domain might originally have been *P5CS* genes that underwent a deletion event, resulting in the loss of the GK or GPR domains. It is important to note that the proline biosynthesis pathway does not seem to have been compromised in these organisms since their genomes contain genes coding for the GK and GPR domains (Supplementary Figure S3).

**FIGURE 1:**
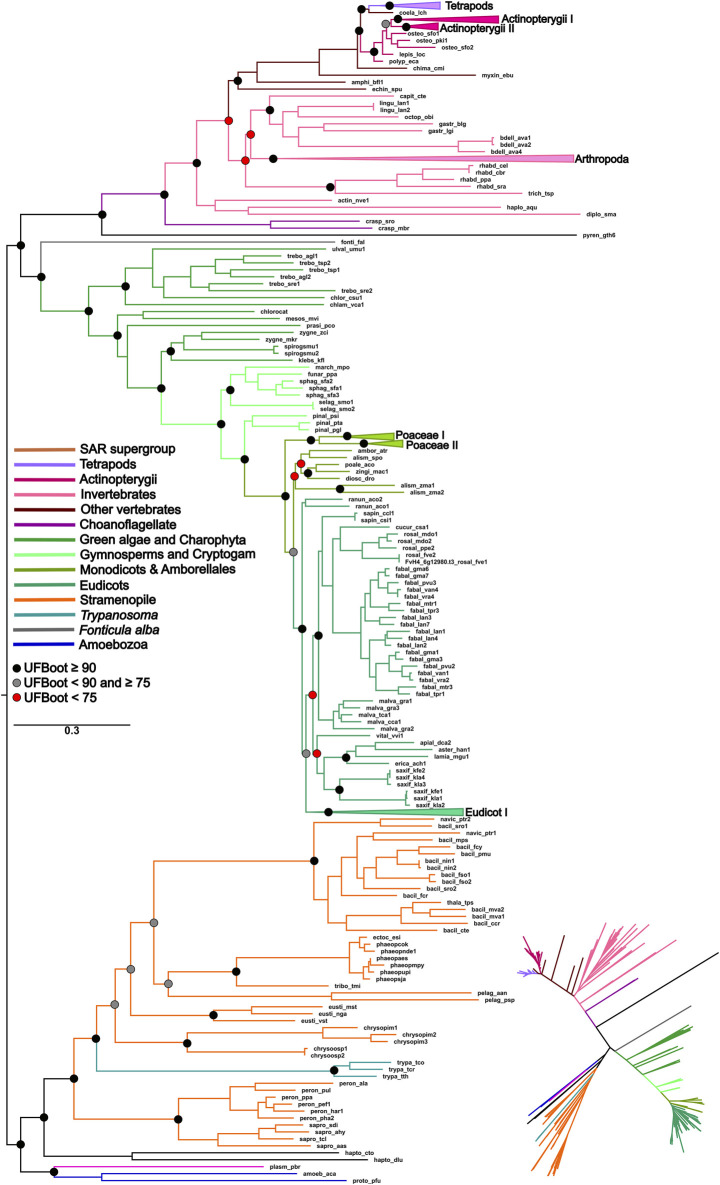
ML phylogeny of the *P5CS* gene. The tree was calculated using the SYM + R7 model. Only ultrafast bootstrap (UFboot) in basal nodes and nodes that define the main taxa are shown. The branches are colored according to the main taxa.

The topology of the ML phylogenetic trees of the *P5CS*, *ProB,* and *ProA* genes generally follows the pattern of the species tree. The phylogenies of *P5CS* showed three main clades, one constituted by plant species, another by animals, and the last by unicellular eukaryote species (e.g., Stramenopiles) (Figure 1). The evolutionary relationship between the subclades is also well supported, with only a few exceptions (e.g., the relationship between the subclades of mammals) (Supplementary Figure S4). The phylogenies of *ProB* and *ProA* are found in the (Supplementary Figures S5, S6).

### 3.2 Origin of *P5CS* and its evolutionary relationships with *ProA* and *ProB* gene

**FIGURE 2:**
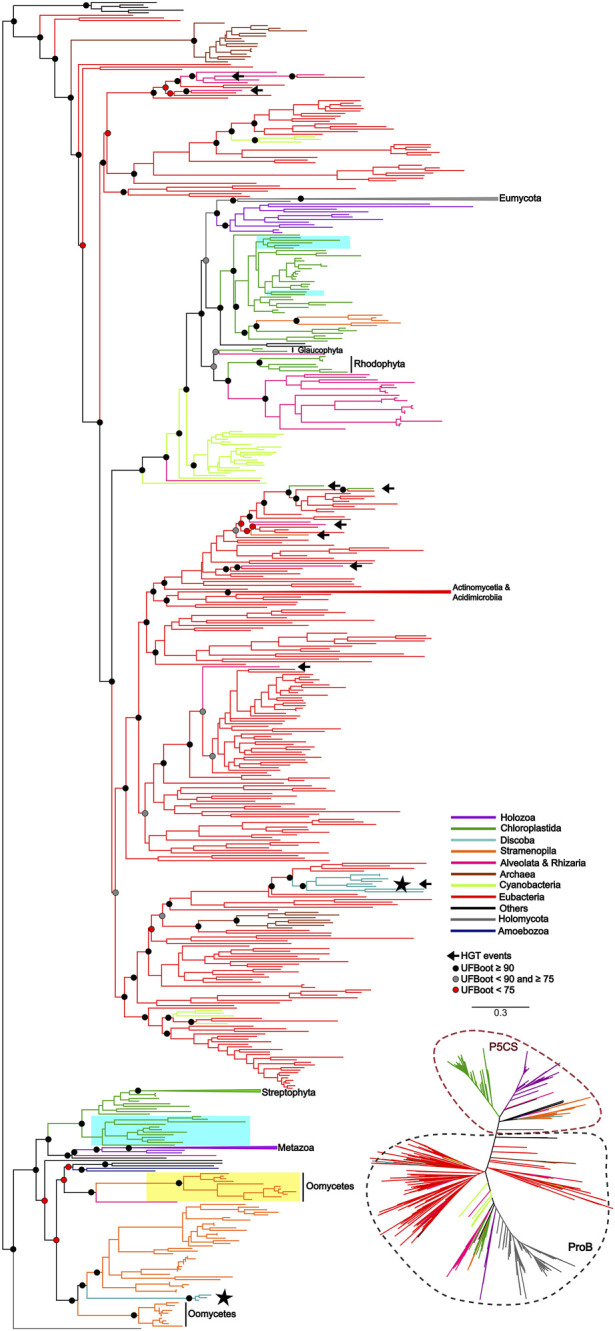
ML tree based on the GK domain of *P5CS* and *ProB* sequences. The tree was calculated using the GTR + F + R10 model. Only ultrafast bootstrap (UFboot) in basal nodes and nodes that define the main taxa are shown. The branches are colored according to the main taxa. The black arrow highlights the potential events of Horizontal gene transfer (HGT) that occurred between prokaryotes to eukaryotes. The black star highlights the sequences of the trypanosomatid species; Highlighted in blue are the genes *P5CS* and *ProB* of the green algae species, that possess both genes in their genome; Highlighted in yellow are the sequences belonging to Oomycetes that have only the GK domain, which we hypothesized to be a “partial *P5CS*”.

**FIGURE 3:**
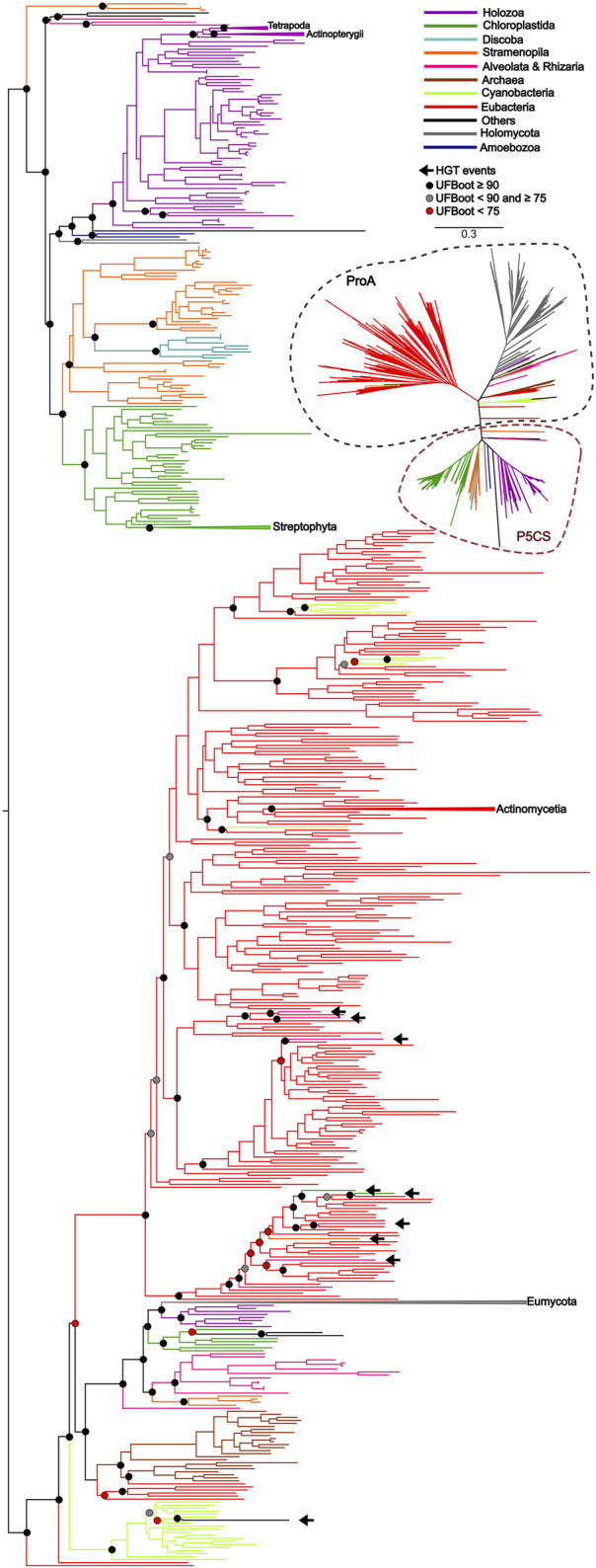
ML tree based on the GPR domain of *P5CS* and *ProA* sequences. The tree was calculated using the GTR + F + R10 model. Only ultrafast bootstrap (UFBoot) in basal nodes and nodes that define the main taxa are shown. The branches are colored according to the main taxa.

To uncover the origin of the *P5CS* gene and understand its evolutionary relationships with *ProB* and *ProA* genes among all living organisms, we constructed phylogenetic trees based on GK and GPR domains separately (Figures 2, 3). Uncollapsed phylogenies can be found in the Supplementary Figures S7, S8. In general, the tree topology was similar to that found within the phylogenies of each isolated gene. Both domain phylogenies formed two main superclades, showing that the *P5CS* gene clustered separately from the *ProB* (Figure 2) and *ProA* (Figure 3) genes. This result may be evidence that the fusion between the *ProA* and *ProB* genes, which gave rise to *P5CS*, occurred only once in the evolutionary history of eukaryotes. Besides, the *P5CS* gene’s origin seems to be an old event in the Tree of Life. This gene is found in early eukaryotic lineages (e.g., Stramenopiles) and sister groups of plants (Charophyta) and animals (Choanoflagellates). These three groups form distinct and unique clades.

Recalling the putative P5CS that lost one of its domains, these sequences were grouped together in the GK and GPR phylogenies in the P5CS clade. This result reinforces the hypothesis that these forty-eight sequences are more closely related to the *P5CS* gene than to the *ProB*/*ProA* genes. One of the events that could explain the loss of one of the domains of the bifunctional enzyme P5CS would be genomic rearrangements. So, we performed synteny analysis around the *P5CS* gene and these supposed “monofunctional P5CS”. Interestingly, for Trypanosomatids, synteny analysis showed that the genomic region of *P5CS* is relatively conserved, and species that have the “monofunctional P5CS” also have the insertion of an upstream gene (Ribosomal protein L3) of the *P5CS*, which may have caused the loss of the GK domain in them. As for algae, the region around the *P5CS* proved to be quite variable between species, and it was difficult to detect any conservation pattern between them.

### 3.3 Duplication and losses of genes

**FIGURE 4:**
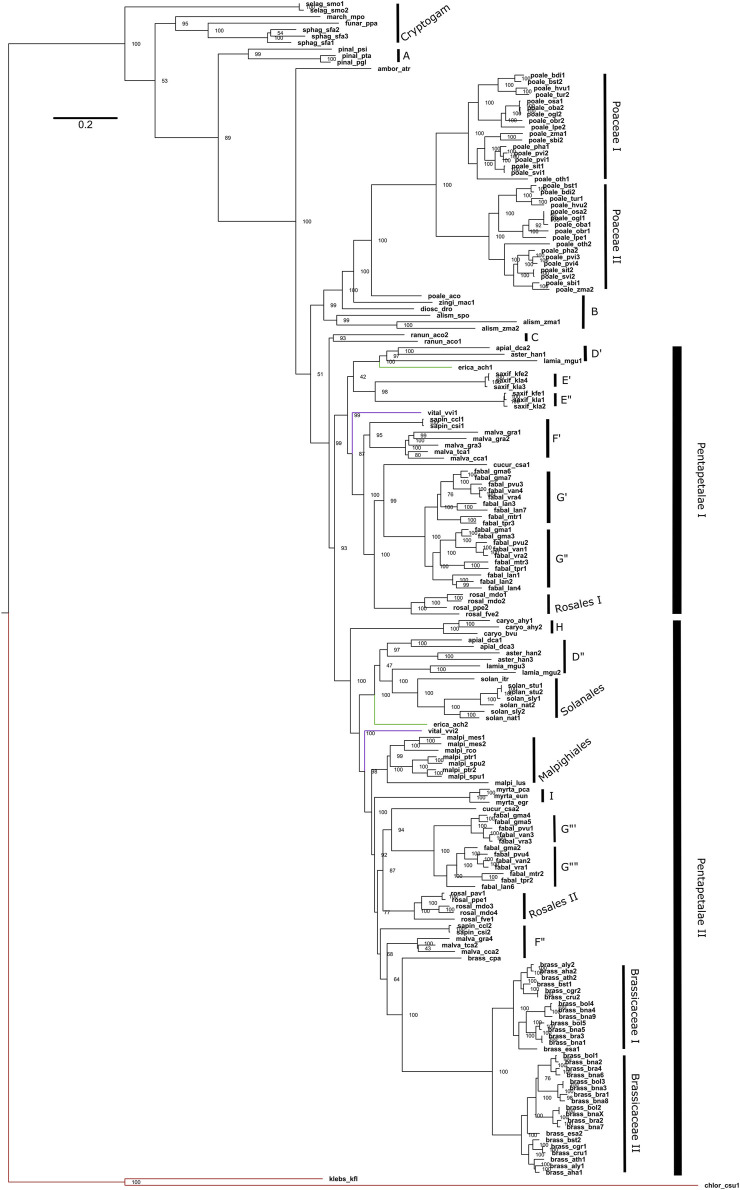
Phylogeny of Maximum Likelihood of the Viridiplantae *P5CS* gene. The tree was obtained using the GTR + F + R5 model and the ultrafast bootstrap (UFBoot) values are shown. The letters used in the phylogeny are representing the sampled orders, being: A (Pinales); B (other Monocots); C (Ranunculaceae); D (Apiaceae, Asteraceae and Phrymaceae); E (Crassulaceae); F (Rutaceae and Malvaceae); G (Fabaceae); H (Amaranthaceae); I (Myrtaceae). The red branch belongs to algae, the green to Actinidiaceae family and the purple branch to the Vitaceae family.

Regarding the duplications in the *P5CS* gene, the animals mostly have a single gene, with the Actinopterygii: Teleostei being the only lineage to present two *P5CS* genes (Figure 2). We found a few other duplications in metazoans, but they are dispersed and unique in the species that have them. In contrast, it is common for plants to have two or more *P5CS* genes, and the pattern found in our phylogeny suggests that several independent gene duplication/loss processes have occurred (Figure 4). The oldest duplication event in Viridiplantae, which we can trace and in which the paralogs remain (in which we can see the typical topology of paralogs genes), seems to have occurred in the ancestor of the Pentapetalae group. Looking at this specific group, we can detect at least four instances in which one of the paralogs was lost, followed later by a new duplication event. These occurrences are observed in Brassicaceae, Crassulaceae, Solanaceae and Salicaceae (Supplementary Figure S9).

For the *ProB*/*ProA* gene, duplications are less common, with a higher prevalence in *ProB* (56 species with duplications) compared to *ProA* (16 species with two or more genes) (Table 2). Duplications found in *ProA* are dispersed, and no evolutionary pattern of duplication is apparent. In contrast, for the *ProB* gene, we observed three taxa with two or more genes: the order of fungi Mucorales (seven species), the family of fungi Saccharomycetaceae (five species), and the order of green algae Mamiellales (three species). However, ProB duplication is not a universal trait in the family of yeasts Saccharomycetaceae, as seven other sampled species from this family possess only a single *ProB* gene.

## 4 Discussion

### 4.1 P5CS origin

P5CS is a bifunctional enzyme encoded by the *P5CS* gene and has originated from the fusion of the *ProB* and *ProA* genes. Previous studies showed that gene fusions are rare, and 31 of the 51 cases analyzed are explained by a single gene fusion event that spread via horizontal gene transfer (HGT) (Yanai et al., 2002). In this study, we hypothesized that the evolutionary history of the *P5CS* is marked by a single gene fusion event, followed by the HGT event. The results found with the phylogenies of the GK and GPR domains showed a clear separation between the bifunctional enzyme and its monofunctional homologs *ProB* and *ProA*. The fact that the *P5CS* gene of all analyzed species forms a clade suggests that the fusion occurred and was fixed only once in the evolutionary history of this gene. This indicates that all species that possess the *P5CS* gene inherited it from a common ancestor in which this single fusion event occurred (Figures 2, 3). Following this logic, if we apply the characteristic “presence of the *P5CS* gene” to the phylogeny of eukaryotes (Burki et al., 2020), we would have the formation of a polyphyletic group. This result highlights a potential role of HGT in spreading the *P5CS* gene among the eukaryotic lineages that possesses this gene. Van Etten and Bhattacharya (2020) provided a comprehensive review of HGT in protists, emphasizing its significance in driving adaptations. They reported that HGT varies from 0.04% to 6.49% among microbial eukaryotes. It is worth emphasizing that the HGT events proposed for the *P5CS* are prior to the multicellularity event, considering that the unicellular ancestors of plants and animals already had the *P5CS*. In addition, the entry of an enzyme into an existing pathway, and possibly existing enzymes, facilitates the establishment of HGT in the genome (Cohen et al., 2011). The work of Ocaña-Pallarès et al. (2019), analyzed the evolutionary history of the genes involved in nitrate assimilation, and the most parsimonious scenario found involves at least seven HGT events among eukaryotic lineages.

Would a scenario in which all eukaryotes directly inherited the *P5CS* gene, with only a few lineages subsequently losing it, be a more parsimonious scenario? The phylogenetic tree revealed that eukaryotes’ *ProB*/*ProA* genes also form a monophyletic group, suggesting that they also inherited them from a single common ancestor. Therefore, in a scenario in which *P5CS* would be the eukaryotic “ancestral gene,” horizontal gene transfer is still the primary process to explain the monophyly found in the *ProB*/*ProA* genes of these eukaryotes. The monophyly of eukaryotic *ProB*/*ProA* is less likely to have occurred by HGT, as the different eukaryotic strains would have had to have received the operon from the same bacterial strain. The eukaryote-eukaryote transfer would also be less probable because related pathway genes are not necessarily linked in eukaryotic genomes. So, possibly LECA (the last eukaryotic common ancestor) had the *ProB* and *ProA* genes. A few examples of organisms sampled present the complete version of *P5CS* and one of the *ProB*/*ProA* genes. This evidence makes us think there would be little or no evolutionary advantage in maintaining monofunctional and fused forms in a genome. Therefore, it would be unlikely that the ancestral lineages that gave rise to current eukaryotes kept both versions of these genes for so long. The phylogenetic tree based on GK domain revealed that some species belonging to Trypanosomatidae family are grouping within the eubacteria clade, suggesting they probably acquired the *ProB* gene from an HGT event (Figure 2).

**FIGURE 5:**
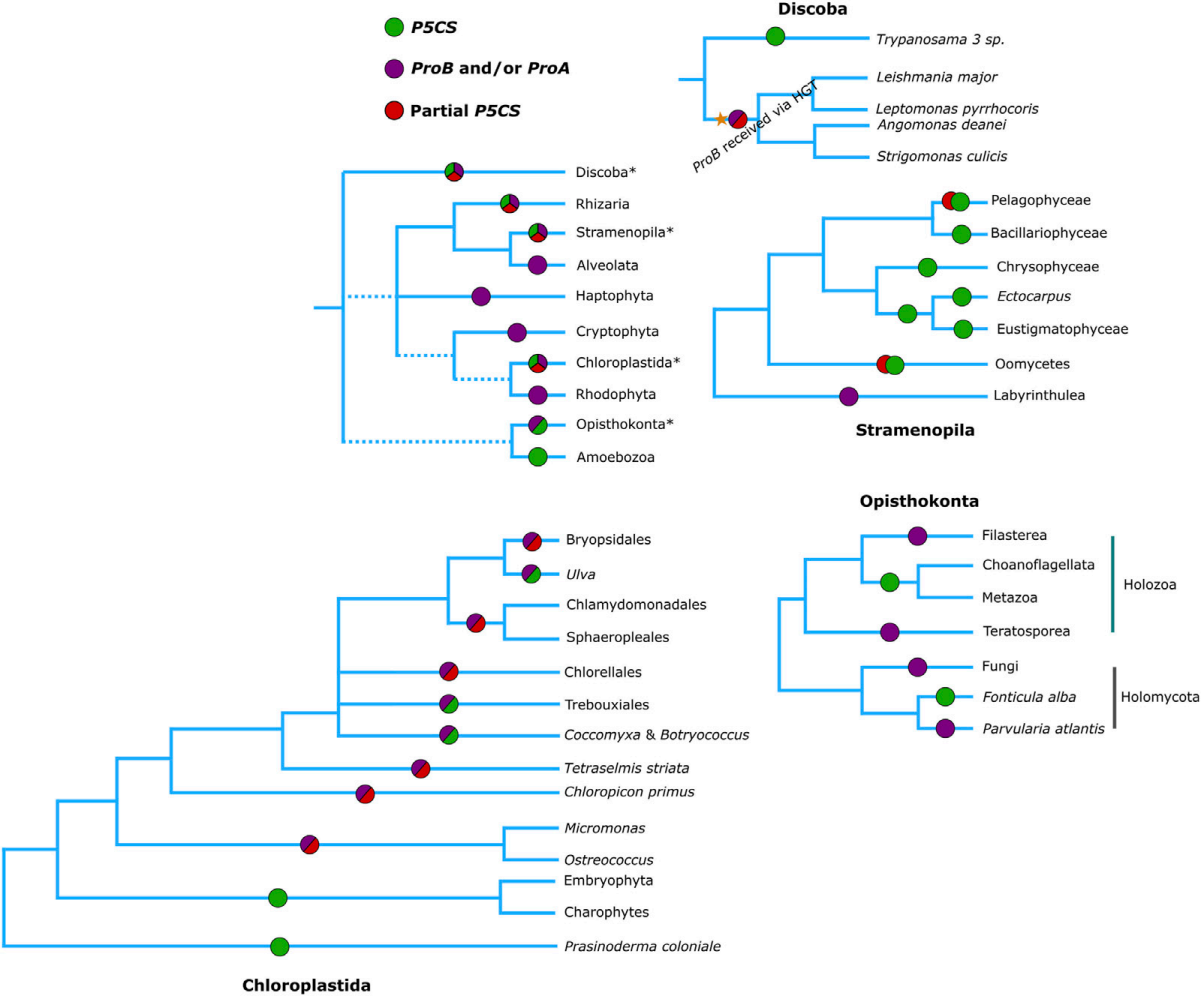
Phylogenetic relationships of eukaryotes (Based on Burki et al., 2020), showing which genes are found in each of the groups. Green circles represent the presence of a complete *P5CS* gene, Red circles the partial *P5CS* gene (with sequence corresponding to only one domain) hypothesized in our work, purple circles represent the presence of *ProA* and/or *ProB* genes.

In view of the results discussed so far, we arrived at the 48 monofunctional sequences belonging to Chlorophytes, Oomycetes and Trypanosomatidae, which were used only in the phylogeny of the domains and were grouped in the superclade P5CS. We hypothesize that these sequences were *P5CS* genes that suffered some deletion in one of their domains, becoming a monofunctional gene again. In the Trypanosomatidae family, we were able to establish a parsimonious evolutionary scenario for our hypothesis since the genus *Trypanosoma* was the first to diverge among the genera analyzed here (Yurchenko et al., 2014). They are the only ones in the family with the complete *P5CS* gene and not having the *ProB* gene. The acquisition of the *ProB* gene via HGT in the Trypanosomatidae lineage must have occurred after the origin of the *Trypanosoma* genus. With the acquisition of the *ProB* gene, the selective pressure under the GK domain of the P5CS enzyme may have been relaxed, allowing the deletion of this portion of the gene (Figures 2, 5). The synteny analysis corroborates this hypothesis since the genomic neighborhood of the GPR domain of these species is similar to that of species that have the complete *P5CS* and an insertion in the N-terminal portion (Supplementary Figure S10). This was also not a deleterious event in oomycetes, as they probably already had another *P5CS* paralog gene (Figure 2), which made it possible to delete the GPR domain in one of the *P5CS* genes. The positioning of oomycetes in phylogeny shows that this deletion occurred in the ancestor before the separation of the orders Saprolegniales and Peronosporales.

For chlorophytes, we do not have such a parsimonious scenario. Our phylogenies do not provide strong evidence for HGT of the *ProB* gene for this group. It is possible that the *ProB* gene was directly inherited from the eukaryotic ancestor. Additionally, it is plausible that the *P5CS* gene was already present in the common ancestor of Viridiplantae, which might have facilitated the loss of the *ProA* gene. And when did the loss of the GK domain of such *P5CS* gene occur? In the lineage of green algae, the deletion process must have occurred twice independently since the first class to diverge was the Mamiellophyceae (here being the first event of loss of the GK domain of its P5CS), the core Chlorophyta being the most derived class (Leliaert et al., 2016), in which the species *C. subellipsoidea* has the complete *P5CS* gene, while the other two species of this class do not, this being the second event of loss of the GK domain of P5CS (Figure 5). As we did not evaluate the functionality of any of the acquired sequences, we cannot rule out the scenario that the GK domain of the P5CS enzyme in algae is not functional since even the species with the complete *P5CS* also has a *ProB* gene. Perhaps, evolution kept the GK domain of Gene *ProB* and not the *P5CS* as functional. Here, the GK-P5CS is just a trace that the algae already had a complete *P5CS* gene. Also, the results of the algae synteny revealed a very dynamic genome around the *P5CS*, and it was not possible to identify any homology in the structure of the genomes with full/partial *P5CS* (Supplementary Figure S11).

### 4.2 Duplication

In plants, it is common to find duplicates for the *P5CS* gene, and in fact, only 20 of the 79 species analyzed in our work have a single *P5CS* (Table 2). Our results, in agreement with a previous study (Turchetto-Zolet et al., 2009), show that the plant duplication events occurred at several independent times. The Fabaceae family has the largest number of *P5CS* paralog genes in our work. Their topology in phylogeny points to a polyploid process in a common ancestor of the collected species. In accordance with this topology, one recent study provides evidence that legumes underwent at least three whole-genome duplications (WGD). One duplication occurred in the ancestor of the family, and the other two occurred independently in the subfamilies Detarioideae and Papilionoideae (Koenen et al., 2020). Our species analyzed, all belonging to a subfamily Papilionoideae, having then undergone two WGD, explaining why *Lupinus angustifolius* and *Glycine max* have more than four *P5CS* paralogues.

So, consulting the literature, we can report that, possibly, the primary source of origin of the paralogs *P5CS* occurred by polyploidy events and not by isolated gene duplications. The oldest duplication we could map in the Pentapetalae may have arisen from the polyploidy event before the asterid-rosid split (Jiao et al., 2012). We can also link to polyploidy events the duplications of *P5CS* that occurred in the ancestral of recent groups, such as those found in the Poaceae family (Levy and Feldman, 2002), and in the Brassicaceae family (Barker et al., 2009). The *Brassica* species analyzed here, which have more than two genes, has a hexaploid ancestor (Schiessl and Mason, 2020). Our phylogeny shows that there must have been a loss of one of the *P5CS* paralogs in the ancestor of the Malpighiales, and that the new duplications occurred two times independently (Figure 4). The literature supports this information and is also linked to polyploids, which shows that the *Salix*/*Populus* clade has a WGD in their ancestor (Koenen et al., 2020) and that *Manihot esculenta* is a paleotetraploid (Bredeson et al., 2016). An origin from a polyploid ancestor is also proposed for the *Kalanchoe* genus, but more rigorous studies and tests on this hypothesis are lacking (Mort et al., 2001).

Functional studies with *A. thaliana* have already demonstrated a certain differentiation between the functions of its two paralogs genes, with *AtP5CS1* being more responsive to stress and *AtP5CS2* being the housekeeping, acting more in the development of the plant (Szèkely et al., 2008). A more recent study reinforced the role of *AtP5CS2* in plant growth and seed germination and that *AtP5CS1* is mainly responsible for proline accumulation in response to salt stress. An interesting result was the osmolarity analysis of the control and knockout plants, which showed that the absence of the *AtP5CS2* gene made the plant more tolerant to salt stress and led to a lower accumulation of sodium ions in the leaves (Funck et al., 2020). In the Poaceae family, works with *Oryza sativa* and *Sorghum bicolor* also showed that their paralogs have different expression patterns in the tissues and that they can play non-redundant roles in plant development (Hur et al., 2004; Su et al., 2011). Our results point to several independent processes of duplication of the *P5CS* gene. The species of Poaceae and Brassicaceae have relatively recent duplications, which probably occurred in the ancestor that gave rise to their families. As suggested by Stiti et al. (2021), we also believe there may be an evolutionary tendency for subfunctionalization between the *P5CS* paralogs of plants. One would have a more significant role in plant development, and the other would act more in the response and prevention of different types of stress.

As already said, plants having two or more *P5CS* genes is quite common, but it is not a rule because some families and species have only one gene. When comparing gene trees with species trees, it is evident that some species previously possessed *P5CS* paralogs but lost them during their evolution, such as Myrtaceae, *Carica papaya*, *Ipomoea triloba*, *Ricinus communis,* and other eudicots. For other species, it is more difficult to track whether they have already had a duplication of the *P5CS* gene or not, as for *Amborella trichopoda*, Pinaceae, *Musa acuminata,* and other monocots. Despite this, the *P5CS* of these species may have evolved distinct mechanisms, like differential alternative splicing forms. Experiments with cotton showed that *P5CS* is one of the genes with alternative differential splicing under salt stress (Zhu et al., 2018). Alternative splicing was also demonstrated in *A. thaliana* by Kesari et al. (2012).

### 4.3 Sequence conservation

Firstly, the subcellular localization of the P5CS enzyme seems to be conserved within kingdoms and different between them. *In silico* analysis of signal peptides showed that 99.4% of plant sequences are predicted to be cytoplasmic, corroborating the functional work of (Funck et al., 2020). While for animals, 74.6% of the sequences were predicted to be mitochondrial in agreement with (Hu et al., 2008). These differences between the enzyme’s locations may denote specificities of function from this pathway between plants and animals. Following this reasoning, we began our analysis with the proline binding sites in the GK domain responsible for inhibiting P5CS via competition. The crystallography of *E. coli* γ-GK revealed the potential residues responsible for the interaction with proline (Marco-Marín et al., 2007). We highlight the glutamate residue, E135 in EcGK (*E. coli*). It is located very close to two important residues for glutamate and proline binding (N134 and D137 in EcGK). Most of the animals have a threonine residue (Supplementary Figure S12A) and are the only ones not to have glutamate in this position (this is a highly conserved residue, with no variation found in the other sequences collected, even when looking at the *ProB* gene). Although the mutation of this residue in *E. coli* (E135A) did not affect the binding sensitivity of proline (Pérez-Arellano et al., 2010), it is interesting to note how the animals P5CS, which are not regulated by proline, are the only ones not to have glutamate at this position.

A recent study unveiled the 3D structure of P5CS from *D. melanogaster* (Zhong et al., 2022). They demonstrated how P5CS forms a filamentous structure called cytoophydium (which has the P5CS tetramers as its basic unit), which is essential for the catalytic activity of P5CS. Two points proved important for the formation and stabilization of this structure. The first site is R124, and the second is F642, using the *D. melanogaster* P5CS as reference. These sites are highly conserved in animals, with no variations found in the 272 sequences collected (Supplementary Figures S12B, C), which raises the hypothesis that P5CS filaments are conserved in the animal lineage, as suggested by Zhong et al. (2022). However, we do not find these amino acids when we look at the homologous sites in plants. In the most critical site for filamentation (Supplementary Figure S12E), we have a gap of two amino acids in plants (only three species do not have this gap). Therefore, the small loop necessary for the interaction between tetramers is likely not formed. In addition, in the second site required for filamentation, the predominant residue in the plant lineage is an Alanine (Supplementary Figure S12D). This residue was even used as a mutation in the study (Zhong et al., 2022), showing that its presence disturbs the formation of the cytoophidium. A new study has shown that P5CS2 from *A. thaliana* also has the ability to form a similar structure (Guo et al., 2023), but using a different molecular mechanism, in line with the structural differences highlighted.

Looking at the conserved sites, residues Gln80 and Gln100 from *E. coli* proved essential for the hydrogen-bond network that links the two active centers in the dimer (Marco-Marín et al., 2007). The function of these residues, probably, is also maintained in the P5CS enzyme, as they are highly conserved sites (Supplementary Figure S12F). This network and N149 are important for the correct positioning of Residue N134, which promotes interaction with the substrate (glutamate) (Supplementary Figure S12A). Catalysis occurs through K10 and K217, using *E. coli* γ-GK as reference, (Supplementary Figure S12G) also requiring D150 (Supplementary Figure S12A), which is a key residue for organizing the active site (Marco-Marín et al., 2007).

For the GPR domain, functional residue data is scarcer. The evaluation of the γ-GPR from *Thermotoga maritima* (Page et al., 2004), showed that this enzyme has a well-known conformation, the Rossmann-like fold. This fold is present in enzymes from the nucleotide and amino acid metabolism (Kamiński et al., 2022). The structure of the *Drosophila* P5CS showed residues R712 and D715 act in the binding of G5P (Zhong et al., 2022). These residues are highly conserved in the sequences collected (Supplementary Figure S12H **)**. The catalytic cysteine, C598 and the neighboring asparagine (N599), in *D. melanogaster* P5CS, are also conserved (Supplementary Figure S12I). However, the 525REE527 loop in the interaction with NAD(P) is less conserved in the γ-GPR enzymes (Supplementary Figure S12K **)** than in the P5CS enzymes (Supplementary Figure S12J).

## 5 Conclusion

In conclusion, our results point to only a single fusion event between the *ProA* and *ProB* genes, which gave rise to the bifunctional form of the P5CS enzyme. Probably, the fusion occurred early in the evolution of eukaryotes and was spread among the ancestors of the plants and animals via HGT. Besides some monofunctional forms found in green algae and Trypanosomatida (GPR domain), and Oomycetes (GK domain), we believe they were originally *P5CS* genes that suffered deletion in one of the domains, and for trypanosomatids, the synteny results support this hypothesis. Our results also suggest that there have been several independent processes of duplication and loss of the *P5CS* gene in plants. In many cases, we have been able to correlate the duplication events with polyploidy events, perhaps the main source of origin of the *P5CS* paralogs in plants.

## Data Availability

The datasets presented in this study can be found in online repositories. The names of the repository/repositories and accession number(s) can be found in the article/Supplementary Material.

## Supplementary material

Supplementary Table S1 Accession number, code used in the study and details of the sequences collected and analyzed.

Supplementary Table S2 Probability of signal sequences via TargetP 2.0.

Supplementary Figure S1 The domains present in the *ProA*, *ProB* and *P5CS* genes. GK (γ-glutamyl kinase); GPR (γ-glutamyl phosphate reductase) and PUA (pseudouridine synthase and archeosine transglycosylase).

Supplementary Figure S2 Alignment used in the phylogenetic analysis of the *P5CS* gene and the GK and GPR domains. The portions without alignment with the *ProB*/*ProA* genes were used only in the analysis of the *P5CS* gene. The arrows show the sites detected under positive selection, the green ones in the *P5CS* of plants and the pink ones in the *P5CS* of the animals. Simplification, using only a sequence of the main groups (*P5CS Homo Sapiens*; P5CS1 and P5CS2 *Arabidopsis thaliana*; *ProB*/*ProA Escheria coli*—proteobacteria; *ProB*/*ProA Saccharomyces cerevisiae*—fungi; *ProB*/*ProA Synechococcus elongatus*—cyanobacteria; *ProB*/*ProA Bacillus cellulosilyticus*—firmicutes).

Supplementary Figure S3 Venn diagram showing the distribution of the *P5CS*, *ProB*, *ProA* genes in the 1028 species analyzed in the study. GK and GPR represent the sequences with a single domain hypothesized as *P5CS* that lost one of the domains.

Supplementary Figure S4 Uncollapsed ML phylogeny of the *P5CS* gene. The UltraFast Bootstrap is represented in the branches. The branches are colored according to the main taxa.

Supplementary Figure S5 ML phylogeny of the *ProB* gene. The tree was calculated using the GTR+F+R10 model and the parameters “-pers 0.2 -nstop 500”. The UltraFast Bootstrap is represented in the branches. The branches are colored according to the main taxa. The black arrow highlights the sequences of eukaryotic species clustered with the bacteria, probably having acquired the *ProB* gene via HGT.

Supplementary Figure S6 ML phylogeny of the *ProA* gene. The tree was calculated using the LG+F+I+R10 model. The UltraFast Bootstrap is represented in the branches. The branches are colored according to the main taxa. The black arrow highlights the sequences of eukaryotic species clustered with the bacteria, probably having acquired the *ProA* gene via HGT.

Supplementary Figure S7 Uncollapsed ML phylogeny of the GK domain. The UltraFast Bootstrap is represented in the branches. The branches are colored according to the main taxa.

Supplementary Figure S8 Uncollapsed ML phylogeny of the GPR domain. The UltraFast Bootstrap is represented in the branches. The branches are colored according to the main taxa.

Supplementary Figure S9 Cladogram of the plant species used in the article, plotted with the events of duplication/loss of the *P5CS* gene that occurred in the evolution of this group. Cladogram generated by timetree.org and the phylogeny used to plot duplication events can be found in the Figure 4. The points marked only as “polyploidy”, are those discussed in the article that have evidence of polyploidy/WGD events.

Supplementary Figure S10 Synteny map around the *P5CS* gene in trypanosomatids. The shaded arrows show the region that would correspond to the complete query, and the fully filled arrow, the region that obtained BLAST results. Trypa_tth (*Trypanosoma theileri*), Trypa_tcr (*Trypanosoma cruzi*), Trypa_tco (*Trypanosoma conorhini*), have the complete *P5CS* and Trypa_lma (*Leishmania major*), Trypa_lpy (*Leptomonas pyrrhocoris*), Trypa_ade (*Angomonas deanei*) have only the GPR domain. Trypa_tth was used as a parameter for searches via BLAST in the analysis, using the four genes upstream to *P5CS* (P-1, P-2, P-3 and P-4) and four downstream (P1, P2, P3 and P4), and also four genes upstream to Ribosome pL3—gene that appeared inserted in the species that underwent horizontal transfer—(Pl3-1, Pl3-2, Pl3-3 and Pl3-4) and four downstream (Pl31, Pl32, Pl33 and Pl34).

Supplementary Figure S11 Synteny map around the *P5CS* gene in the green algae. Chlor_csu (*Coccomyxa subellipsoidea*) and Chlor_bbr (*Botryococcus braunii*) have the complete *P5CS* gene. Chlor_czo (*Chromochloris zofingiensis*), Mamie_msp (Micromonas sp. RCC299), Mamie_mpu (*Micromonas pusilla*), Mamie_olu (*Ostreococcus lucimarinus*), Chlam_cre (*Chlamydomonas reinhardtii*), Chlam_vca (*Volvox carteri*), have only the GPR domain. Chlor_csu was used as a reference for searches via BLAST in the analysis, using the four genes upstream to *P5CS* (P-1, P-2, P-3 and P-4) and four downstream (P1, P2, P3 and P4), and Mamie_msp was used as a reference for the upstream and downstream genes of the *ProB1* and *ProB2* genes.

Supplementary Figure S12 Logo of the main regions involved in the catalytic activity of the GK and GPR domains. The brow arrows highlight the main residues discussed in the manuscript, and the black arrows highlight the residues involved in the hydrogen bond chain that links the two catalytic centers in the dimer. EcGK, AtP5CS1, DmP5CS, EcGPR are the residue numbers for the γ-GK enzyme from *Escherichia coli*, *P5CS1* from *Arabidopsis thaliana*, *P5CS* from *Drosophila melanogaster* and γ-GPR from *E. coli*, respectively. **(A)** Logo from all sequences containing the GK domain analyzed in this study; the purple arrow marks the conserved glutamate residue in the γ-GKs and in the non-animal *P5CS*, the threonine present in the logo is due to the *P5CS* of the animal species. **(B–E)** Logo from the regions involved in the filamentation of the *P5CS* in **(D)**. melanogaster, the arrows points to essential residues for the filamentation; **(B, C)** logo only from the 275 animal *P5CS* utilized in our study, with no variation in the essential residues; **(D, E)** logo only from the 180 plant *P5CS* utilized in our study. **(F, G)** Logo made with all the sequences containing the GK domain analyzed in this study, **(F)** showing the conservation of the glutamines involved in the hydrogen bond; **(G)** highlighting the conservation of the catalytic lysines. **(H, I)** Logo made with all the sequences containing the GPR domain analyzed in this study; **(H)** highlighting the residues involved in binding to the substrate (Glutamyl-5-phosphate); **(I)** highlighting the catalytic cysteine. **(J)** Logo made of all *P5CS* analyzed, highlighting the conservation of the REE loop, which interacts with NAD(P). **(K)** Logo made of all the γ-GPR, highlighting the lower conservation of the REE loop compared to the *P5CS*.
