# Supplementary figures and images for: Unraveling the evolutionary origin of the P5CS gene: a story of gene fusion and horizontal transfer
Source: Front Mol Biosci. 2024 Apr 17;11:1341684. doi: 10.3389/fmolb.2024.1341684 (PMC11061531; doi:10.3389/fmolb.2024.1341684)

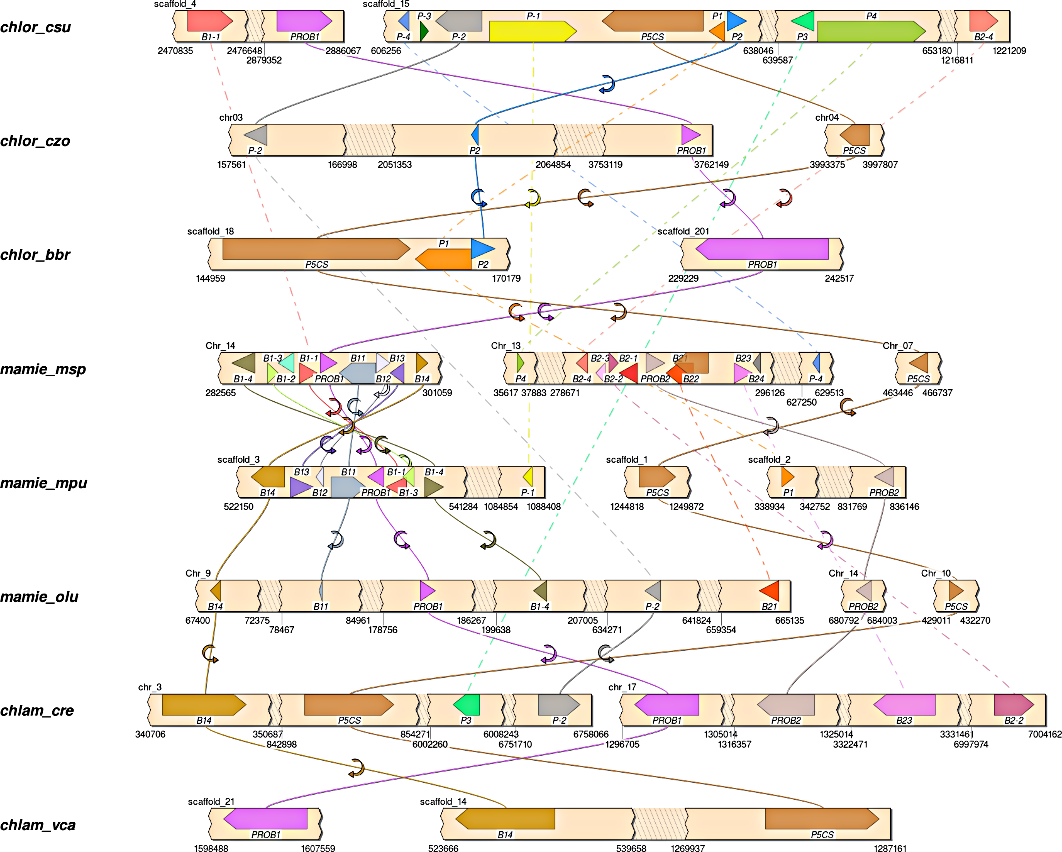

Supplement: Supplementary file 2 [file Image11.PNG]

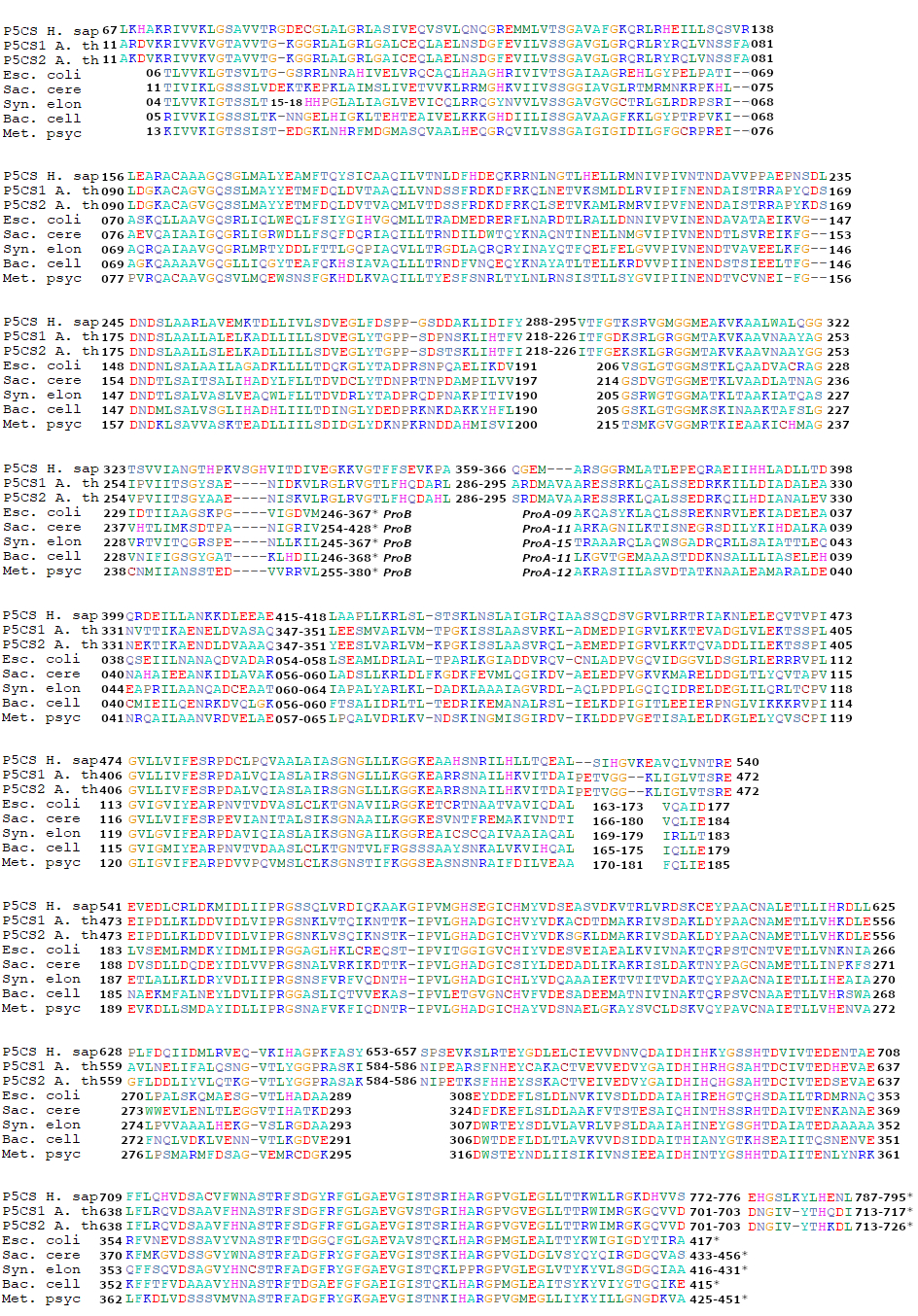

Supplement: Supplementary file 7 [file Image2.JPEG]

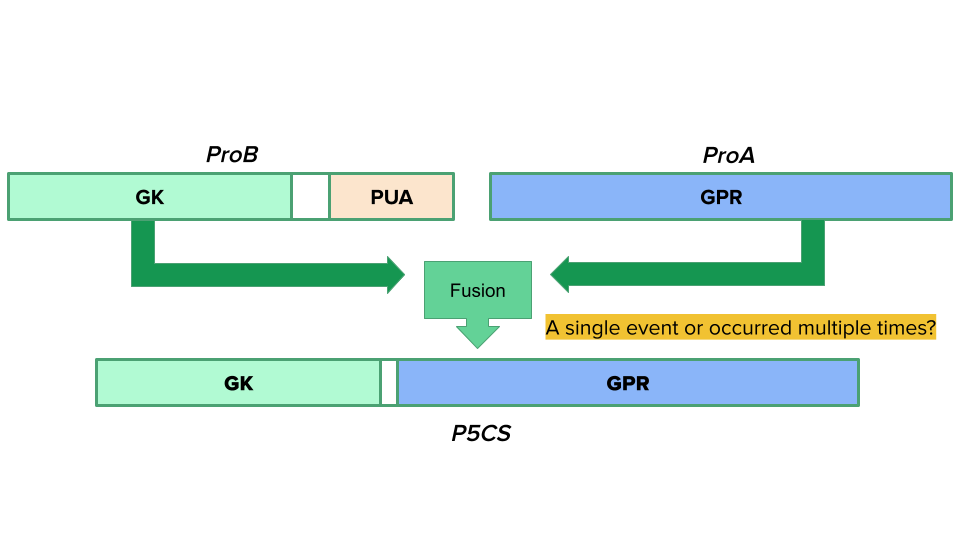

Supplement: Supplementary file 10 [file Image1.PNG]

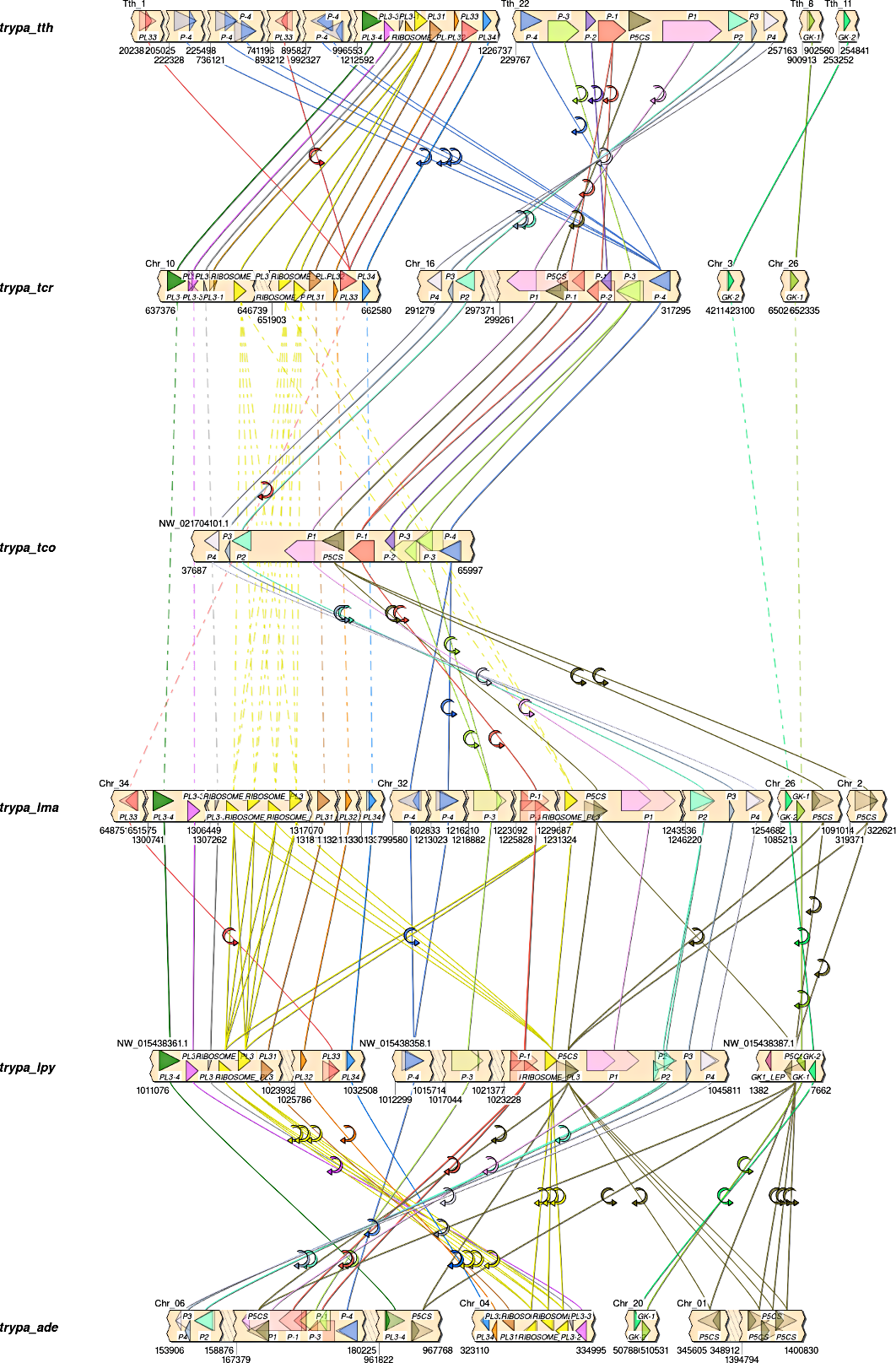

Supplement: Supplementary file 14 [file Image10.PNG]
